# Determinants of fruit and vegetable consumption among Saudi adults: an extended Theory of Planned Behavior approach

**DOI:** 10.3389/fpubh.2025.1593625

**Published:** 2025-08-19

**Authors:** Eman A. Abduljawad, Buthaina M. Aljehany, Haya Aljadani, Howeida Abusalih

**Affiliations:** ^1^Food and Nutrition Department, Human Sciences and Design Faculty, King Abdulaziz University, Jeddah, Saudi Arabia; ^2^Department of Health Sciences, College of Health and Rehabilitation Sciences, Princess Nourah bint Abdulrahman University, Riyadh, Saudi Arabia

**Keywords:** fruits and vegetables, consumption, Theory of Planned Behavior, Saudi Arabia, adults

## Abstract

**Aim:**

This study examines the determinants of fruit and vegetable consumption among Saudi adults using an extended Theory of Planned Behavior (TPB) model. The model incorporates core TPB constructs—attitudes, subjective norms, and perceived behavioral control (PBC)—along with sociodemographic, behavioral, and knowledge-related factors.

**Methods:**

A cross sectional study was conducted on a sample of adult Saudis (*n* = 471). Data were analyzed using structural equation modeling, comparing a basic TPB model with an extended model that included additional predictors such as knowledge about World Health Organization (WHO) dietary recommendations, diet, family meals, and physical activity.

**Results:**

PBC has is a strong predictor of behavior compared to intention. For intention, attitude (unstandardized coefficients [coeff] = 0.29, standard error [se] = 0.13, *p* = 0.025), subjective norm (coeff = 0.37, se = 0.11, *p* = 0.001), and PBC (coeff = 1.29, se = 0.17, *p* = 0.000) are all significant predictors. The extended model explained slightly more variance in behavior (*R*^2^ = 0.45) and intention (*R*^2^ = 0.78) compared to the basic model (behavior: *R*^2^ = 0.40; intention: *R*^2^ = 0.74), highlighting the added value of these factors. PBC emerged as the strongest predictor of behavior (coeff = 0.71, se = 0.26, *p* = 0.006), while knowledge (coeff = 0.29, se = 0.05, *p* < 0.001) and family meals (coeff = 0.19, se = 0.05, *p* < 0.001) significantly predicted attitudes. Moderate physical activity was associated with subjective norms (coeff = 0.08, se = 0.03, *p* = 0.009), suggesting a synergistic relationship between physical activity and dietary behavior.

**Conclusion:**

These findings underscore the importance of addressing both psychological and practical factors in interventions to promote fruit and vegetable consumption among Saudi population, with a focus on enhancing self-efficacy, leveraging family dynamics, and integrating physical activity promotion.

## 1 Introduction

It is well established that the consumption of fruits and vegetables is widely recognized as a cornerstone of a healthy diet, playing a critical role in preventing chronic diseases such as obesity, cardiovascular diseases, diabetes, hypertension, coronary heart disease, and stroke World Health Organization (WHO) (1, 2), with recent meta-analyses demonstrating dose-dependent mortality reductions (3). Beyond chronic diseases prevention, adequate intake of fruits and vegetables has been associated with the prevention of weight gain, the delay of geriatric conditions (4), modification the gut microbiota composition and reducing the pro-inflammatory response (5). Considering this scientific evidence, the WHO and the Food and Agriculture Organization (FAO) advise a minimum daily consumption of 400 grams of fruits and vegetables per person, excluding starchy tubers (6), recently reaffirmed in updated global guidelines (40). This guideline is frequently promoted through public health campaigns like the “Five-a-Day” initiative, which advocates for the daily intake of at least five servings of fruits and vegetables (7). Despite these widely recognized recommendations, global compliance with dietary guidelines remains inadequate, as many populations do not achieve the advised levels of intake (2). Emerging solutions include food system approaches (8), digital interventions (9), and fiscal policies (10) to address these persistent challenges.

To better understand and encourage healthier eating habits, researchers have increasingly relied on psychological and behavioral theories, such as the Theory of Planned Behavior (TPB) (11). According to the TPB, behavior is shaped by three core constructs: attitudes, subjective norms, and perceived behavioral control (PBC). Attitudes represent an individual’s positive or negative evaluation of a behavior, subjective norms reflect the perceived social pressure to engage in the behavior, and PBC refers to the perceived ease or difficulty of performing the behavior (11). The TPB has been extensively used to predict and analyze health-related behaviors, including dietary choices, across diverse cultural settings (12, 13, 39). Recent meta-analyses confirm its robustness in explaining dietary intentions, particularly for fruit and vegetable consumption (14, 15). For instance, research has shown that TPB constructs explain a significant portion of the variance in both the intention to consume fruits and vegetables and actual consumption levels (12). Moreover, interventions based on the TPB have proven effective in increasing fruit and vegetable intake while positively impacting TPB-related factors such as attitudes and perceived control (Kothe et al., 2012). In addition to the constructs of the Theory of Planned Behavior (TPB), sociodemographic and behavioral factors—such as age, gender, education, income, nutrition knowledge, family habits, and physical activity—have also been identified as significant predictors of dietary behavior (16–19). For example, studies have found that greater nutrition knowledge and regular family meals are positively associated with higher fruit and vegetable consumption (18, 20). Similarly, physical activity has been shown to correlate with healthier dietary patterns, indicating a synergistic relationship between these two health-promoting behaviors (17).

In Saudi Arabia, the prevalence of diet-related non-communicable diseases has surged in recent years, driven by rapid urbanization, increasingly sedentary lifestyles, and a shift toward Westernized dietary patterns (21, 22). National health surveys highlight a concerning trend: 97.4% of Saudi adults consume fewer than five servings of fruits and vegetables per day, with only 2.6% meeting the recommended intake (23). More recent data from Riyadh further emphasize this issue, revealing that 91.6% of participants consume fewer than two servings daily (24). These alarming statistics underscore the urgent need to address dietary behaviors in the country. Research has identified several factors influencing fruit and vegetable consumption among the Saudi population, including fitness consciousness, self-efficacy, perceived benefits and barriers to healthy eating, and meal planning (25–27). However, despite the widespread application of the TPB in understanding dietary choices, few studies have explored its utility in the Saudi context, where cultural and social norms may uniquely determine dietary practices (28). This study aims to address this gap by examining the factors influencing fruit and vegetable consumption among adult Saudis using an extended TPB model. By incorporating sociodemographic factors, behavioral variables, and knowledge related to fruit and vegetable consumption, the study seeks to provide a comprehensive understanding of the determinants of this dietary behavior in the Saudi population. The findings will contribute to the development of culturally tailored interventions to promote healthy eating habits, with a particular focus on increasing fruit and vegetable consumption. Specifically, the study proposes several hypotheses grounded in the TPB: (1) a positive attitude will significantly predict the intention to consume fruits and vegetables, (2) subjective norms will play a significant role in shaping these intentions, (3) PBC will significantly predict both the intention to consume fruits and vegetables and the actual behavior, (4) intention will be a strong predictor of actual fruit and vegetable consumption, and (5) lifestyle behaviors, knowledge related to fruit and vegetable consumption, and sociodemographic factors will be associated with the PBC construct.

## 2 Methods

### 2.1 Study population and study design

This study adopted a cross-sectional design and was carried out in Saudi Arabia, covering the five regions (Northern, South, East, Western and Middle). Data was collected over 6 months from May to October 2024. To be included in the study, the participant should be a Saudi citizen, living in Saudi Arabia during the time of the study, and adult aged from 18 to 59 years old. Adults aged 60 years and above were excluded from the study due to significant physiological, psychological, and behavioral differences in dietary patterns and health determinants compared to younger adults (29). In addition, this age group might introduce heterogeneity that may confound the applicability of the TPB model, as older adults tend to rely less on intention and more on habitual behavior and environmental factors when it comes to dietary choices (14, 30). Pregnant or lactating women, individuals on special diets, or those with chronic diseases affecting their fruit and vegetable intake were also excluded from the study.

The study was conducted after obtaining the ethical approval granted by the Institutional Review Board (IRB) of King Abdulaziz University (IRB Log number 15–24).

### 2.2 Sampling technique and sample size calculation

Due to the nature of online distribution, a non-probability convenience sampling technique was employed. Although El Bcheraoui et al. (23) reported that 97.4% of Saudi adults consumed fewer than five servings of fruits and vegetables per day, based on data from the 2013 Saudi Health Interview Survey, this information is outdated and may not accurately reflect current consumption patterns. Therefore, in the absence of current and specific national data, a conservative estimate of 50% prevalence was used. This standard approach is widely accepted when the actual population proportion is uncertain or outdated (31), as it yields to the maximum sample size and statistical power. The adult population of Saudi Arabia was estimated at approximately 13 million, as reported in The Saudi Census (45). The sample size was calculated using the following standard formula:


$$n=Z^{2}\times p(1-p)/d^{2},$$

where:

- *n* = required sample size
- *Z* = Z-score corresponding to the desired confidence level (1.96 for 95%)
- *p* = estimated proportion (set at 0.50 to maximize sample size)
- *d* = margin of error (set at 0.05)

Thus, an initial target of 385 participants was determined. To account for potential challenges such as incomplete submissions or non-responses, an extra 10% was included, raising the minimum required sample size to 422. Ultimately, the study successfully recruited 471 participants.

### 2.3 Data collection

Data were collected through an online, self-administered questionnaire hosted on Google Forms. Participants were recruited through the support of the Scientific Research Committee at King Abdulaziz University. This committee distributed a formal email to all staff and students, which included the study objective and a link to the online questionnaire, inviting them to participate. Additionally, the email encouraged recipients to share the link with their social networks via platforms such as Twitter, Telegram, and WhatsApp. Upon accessing the survey, participants were presented with an informed consent page. Only those who consented were able to proceed to complete the questionnaire. Participant were also informed of the study’s objectives, the voluntary nature of their participation, and their right to withdraw at any time without consequences. Confidentiality was strictly maintained, and no personally identifiable information was collected. The data gathered were used exclusively for research purposes. The questionnaire was provided in Arabic, the native language of the participants, to ensure clarity and ease of understanding.

### 2.4 Instrument for data collection

A structured questionnaire was used in the study and was divided into three distinct sections:

**Section 1—General Information:** This segment gathered general details about the participants, such as their age, gender, marital status, educational background, region of residence, and monthly income in Saudi Riyals (SAR). It also included questions on height (m) and weight (kg) These anthropometric parameters were used to calculate the body mass index (kg/m^2^).

**Section 2—Assessment of Nutrition Knowledge:** One targeted question was included to evaluate participants knowledge related to the recommended daily intake (5 portions) of fruits and vegetables, as outlined by the Saudi Ministry of Health (46).

**Section 3—Evaluation of Lifestyle Behaviors:** This section explored participants’ overall lifestyle habits through a series of questions covering aspects such as physical activity levels, family meal-sharing practices, the description of their diet (healthy, balanced, calorie content), and their involvement in purchasing and preparing fruits and vegetables.

**Section 4—TPB Constructs:** The study was grounded in Ajzen’s conceptual framework for developing a TPB questionnaire (11, 47). The Target, Action, Context, and Time (TACT) strategy was employed to define the behavior under investigation, which was “consuming 5 servings of fruits and vegetables daily over the next week.” The TACT elements were determined in accordance with the recommendations of the Saudi Arabian Ministry of Health. Following the WHO guidelines, starchy roots such as potatoes and sweet potatoes were excluded from the vegetable category. Attitude was measured using four semantic differential scales. Participants were asked to rate statements such as, “E*ating at least 5 servings of fruits and vegetables daily next week is very bad/bad/neutral /good/very good*,” “*Eating at least 5 servings of fruits and vegetables daily next week is not all pleasant/not pleasant/neutral/pleasant/very pleasant*,” “*Eating at least 5 servings of fruits and vegetables daily next week is very unhealthy/unhealthy/neutral/healthy/very healthy*,” and “*Eating at least 5 servings of fruits and vegetables daily next week is very difficult to digest/difficult to digest/neutral/easy to digest/very easy to digest*.” Subjective Norms were assessed through two items: “*My family expects me to eat 5 servings of fruits and vegetables per day next week*” and “*My friends expect me to eat 5 servings of fruits and vegetables per day next week*.” PBC was measured using two items: “*I think that eating 5 servings of fruits and vegetables daily next week is something possible*” and “*I am sure that I can eat 5 servings of fruits and vegetables per day next week*.” Intention was evaluated using three items: “*I intend to eat 5 servings of fruits and vegetables per day next week*,” “*I am sure to eat 5 servings of fruits and vegetables per day next week*,” and “*My aim is to eat 5 servings of fruits and vegetables per day next week*.” Behavior was assessed by measuring the actual consumption of 5 servings of fruits and vegetables daily. Two questions were used: “How many servings of fruits and vegetables have you eaten daily in the last week?” and “How many times have you eaten 5 servings of fruits and vegetables daily in the last week?”

The items of the TPB were assessed using a 5-points Likert scale. Attitude toward the behavior was assessed with four differentials. Two measures of subjective norms were used and PBC was measured with two items. Three items were used to assess behavioral intention. Two items were employed to measure the behavior.

### 2.5 Validity

After finalizing the questionnaire, it was reviewed by a panel of 11 experts specializing in nutrition, public health, and psychology to assess its face validity, following the guidelines outlined by Mokkink et al. (41). Content validity aims to ensure that the questionnaire adequately covers the construct of interest and is conceptually appropriate. This process requires the input of subject-matter experts who can judge clarity, and comprehensiveness of each item based on theoretical and practical knowledge, and the estimated time required to complete the survey (32, 42). Hence, expert validation is considered the gold standard in assessing content validity. The results indicated that 88.9% of the experts considered the content comprehensive, 88.9% found the questions easy to understand, and the average completion time was 8.9 ± 1.4 min. Additionally, the experts were invited to provide feedback on the questionnaire’s usability, identifying any potential ambiguities in wording or content that might require refinement, in accordance with the recommendations of Janssens et al. (43). Their suggestions were incorporated to improve the instrument’s validity and user-friendliness.

Content validity was also evaluated alongside face validity. The Individual Content Validity Index (I-CVI) for each item ranged between 0.82 and 1, with a 97.1% agreement rate among experts. The Scale Content Validity Index (S-CVI)/Average was calculated at 93.3%, while the S-CVI/Universal Agreement stood at 77.4%. These metrics demonstrate the questionnaire’s strong content validity, confirming its effectiveness as a reliable measurement tool.

### 2.6 Internal consistency reliability

The reliability testing consisted in measuring the internal consistency by calculating the Cronbach’s alpha. It was performed on individuals who are representative of the actual study population. This is because the purpose is to assess how consistently the respondents interpret and respond to the items over time or within the same measurement, which would not be generalizable if only experts (who are not the target audience) were used (33, 44). The internal consistency of the scales (Cronbach’s alpha) suggests that the scales are reasonably homogenous.

### 2.7 Statistical analysis

All data analyses were conducted using the R software. Descriptive data were presented as mean ± standard deviation, % and frequencies. A structural equation modeling (SEM) approach was used to test the research hypotheses 1–4. The model fit was assessed with Chi-square (*χ*^2^), comparative fit index (CFI), the Tucker-Lewis Index (TLI), and root mean square error of approximation (RMSEA), and standardized root mean square residual (SRMR), and the coefficient of determination (*R*^2^) was used to measure the explained variance of the endogenous variables (intention and behavior). An adequate model fit is obtained when the CFI and TLI are >0.90 and the RMSEA and SRMR <0.08. The models were estimated using the Maximum Likelihood estimator. To test hypothesis 5, extension of the model was performed by including the lifestyle behaviors, knowledge, sociodemographic factors, in addition to the TPB construct. A bivariate Pearson test were also conducted in a separate analysis than SEM to assess the correlation of the studied parameters. Significance was set at a *p*-value <0.05.

## 3 Results

### 3.1 Descriptive analysis

The sociodemographic characteristics and lifestyle habits of a studied population are summarized in Table 1. The average age of participants is 34.53 ± 13.86 years, with a majority being female (74.58%). Most participants fall within the normal BMI range (43.56% with BMI 18.5–24.9), though a significant portion are overweight (25.95%) or obese (23.11%). Over half of the population was not married (53.18%), and the majority had a bachelor’s degree or higher (88.99%). Geographically, most participants originated from the Western region (62.50%). Income levels were relatively evenly distributed, with the largest group earning 11,000–20,000 monthly (34.32%).

**Table 1:** Sociodemographic characteristics of the studied population.

Studied parameters	Total
	*N* = 472
**Age**	34.53 ± 13.86
**Gender**	
Female	352.00 (74.58%)
Male	113.00 (23.94%)
**BMI (kg/m** ^**2**^ **)**	
<18.5	34.00 (7.61%)
18.5–24.9	196.00 (43.56%)
25–29.9	116.00 (25.95%)
>30	104.00 (23.11%)
**Marital status**	
Married	215.00 (45.55%)
Not married	251.00 (53.18%)
**Educational level**	
Secondary/higher	44.00 (9.32%)
Bachelor/diploma	239.00 (50.64%)
Postgraduate	181.00 (38.35%)
**Place origin**	
Northern	11.00 (2.33%)
Southern	22.00 (4.66%)
Middle	129.00 (27.33%)
Eastern	7.00 (1.48%)
Western	295.00 (62.50%)
**Monthly income (Saudi Riyals)**	
<5,000	53.00 (11.23%)
5,000–10,000	134.00 (28.39%)
11,000–20,000	162.00 (34.32%)
>20,000	115.00 (24.36%)
**Knowledge regarding the WHO daily dietary consumption recommendations**
<3 servings	140.00 (29.66%)
3 servings	183.00 (38.77%)
4 servings	54.00 (11.44%)
5 servings	72.00 (15.25%)
≥6 servings	18.00 (3.81%)
**Family meals**	
Never	24.00 (5.08%)
1–2/day	89.00 (18.86%)
3–4/day	76.00 (16.10%)
5–6/day	59.00 (12.50%)
Daily	218.00 (46.19%)
**Fruits and vegetables purchasing**	
Never	15.00 (3.18%)
Rare	44.00 (9.32%)
Sometimes	114.00 (24.15%)
Often	140.00 (29.66%)
Always	154.00 (32.63%)
**Fruits and vegetables cooking/preparing**	
Never	52.00 (11.02%)
Rare	62.00 (13.14%)
Sometimes	105.00 (22.25%)
Often	119.00 (25.21%)
Always	127.00 (26.91%)
**How healthy the diet is:**	
Very unhealthy	13.00 (2.75%)
Unhealthy	65.00 (13.77%)
Neutral	211.00 (44.70%)
Healthy	159.00 (33.69%)
Very healthy	18.00 (3.81%)
**How balanced the diet is:**	
Very unbalanced	13.00 (2.75%)
Unbalanced	100.00 (21.19%)
Neutral	152.00 (32.20%)
Balanced	187.00 (39.62%)
Very balanced	17.00 (3.60%)
**How caloric the diet is**:	
Very low caloric	9.00 (1.91%)
Low caloric	62.00 (13.14%)
Neutral	216.00 (45.76%)
High caloric	151.00 (31.99%)
Very high caloric	30.00 (6.36%)
**Intense physical activity**	
Never	277.00 (60.48%)
1 time/month	1.00 (0.22%)
1 time/week	42.00 (9.17%)
2–3 times/week	88.00 (19.21%)
4–6 times/week	38.00 (8.30%)
At least 1 time/day	12.00 (2.54%)
**Moderate physical activity**	
Never	162.00 (34.32%)
1 time/month	2.00 (0.44%)
1 time/week	59.00 (13.08%)
2–3 times/week	97.00 (20.55%)
4–6 times/week	79.00 (16.74%)
At least 1 time/day	52.00 (11.53%)
**Light physical activity**	
Never	143.00 (30.30%)
1 time/month	1.00 (0.22%)
1 time/week	53.00 (11.23%)
2–3 times/week	104.00 (22.03%)
4–6 times/week	80.00 (16.95%)
At least 1 time/day	81.00 (17.53%)
**Any type of physical activity leading to increased heart beats**
Never	144.00 (30.51%)
Rare	135.00 (28.60%)
Sometimes	116.00 (24.58%)
Often	65.00 (13.77%)
Always	12.00 (2.54%)

Regarding dietary habits, 38.77% of participants had moderate knowledge of WHO dietary recommendations, and nearly half reported having family meals daily (46.19%). A significant portion frequently purchased (62.29% often/always) and prepared (52.12% often/always) fruits and vegetables. Dietary intake was described as moderately healthy (44.70% middle, 33.69% healthy) and balanced (32.20% middle, 39.62% balanced), with calorie intake mostly moderate (45.76%). Physical activity levels were generally low, with 60.48% never engaging in intense activity and 34.32% never engaging in moderate activity. Light physical activity was more common, though 30.30% still report never participating. Overall, the findings indicate moderate awareness of fruit and vegetable consumption but low levels of physical activity.

Table 2 presents the reliability (Cronbach’s alpha), mean scores, and standard deviations for various constructs related to the consumption of five servings of vegetables and fruits per day. The Cronbach’s alpha values indicate good to excellent internal consistency for all constructs, ranging from 0.72 (Behavior) to 0.92 (Intention). Participants generally held positive attitudes toward consuming five servings of vegetables and fruits daily, with the highest mean score for the item “healthy” (4.35 ± 0.79). Subjective norms showed moderate agreement, with family expectations (3.50 ± 1.08) slightly higher than friends’ expectations (3.25 ± 1.05). PBC was also moderately high, with participants expressing confidence in their ability to consume the recommended servings (3.68 ± 0.94). Intentions to consume five servings were moderately strong, with mean scores ranging from 3.25 ± 1.09 to 3.56 ± 1.06. In terms of actual behavior, participants reported consuming an average of 3.27 ± 0.93 servings daily in the past week, with a frequency of consumption averaging 2.42 ± 1.23.

**Table 2:** Constructs Cronbach’s alpha, mean scores, and standard deviations.

Construct items	Alpha	Mean ± SD
**Attitude**	0.77	
Eating five servings of vegetables and fruits per day next week is:		4.20 ± 0.84
Good		3.93 ± 0.90
Pleasant		4.35 ± 0.79
Healthy		4.07 ± 0.87
Easiness to digest		
**Subjective norm**	0.83	
My family expects me to eat five servings of vegetables and fruits per day next week		3.50 ± 1.08
My friends expect me to eat five servings of vegetables and fruits per day next week		3.25 ± 1.05
**Perceived behavioral control**	0.81	
I think that eating five servings of vegetables and fruits per day next week is possible		3.56 ± 1.02
I am sure I can eat five servings of vegetables and fruits per day next week		3.68 ± 0.94
**Intention**	0.92	
I intend to eat five servings of vegetables and fruits per day next week		3.56 ± 1.06
I am sure to eat five servings of vegetables and fruits per day next week		3.25 ± 1.09
My aim is to eat five servings of vegetables and fruits per day next week		3.55 ± 1.08
**Behavior**	0.72	
Number of servings daily last week		3.27 ± 0.93
Frequency of consumption		2.42 ± 1.23

The frequency of consumption is measured by the following item: “How many days have been eaten five servings of vegetables and fruits last week: 1 = I did not eat, 2 = 1–2 days, 3 = 3–4 days, 4 = 5–6 days, and 5 = daily.

### 3.2 Predicting fruits and vegetables consumption

Table 3 presents the results of the TPB model, including unstandardized coefficients (coeff), standard errors (se), standardized coefficients (std), and *p*-values for predicting behavior and intention. The model explains 40% of the variance in behavior and 74% of the variance in intention. For behavior, both intention (coeff = 0.16, se = 0.06, *p* = 0.012) and PBC (coeff = 0.54, se = 0.14, *p* = 0.000) are significant predictors. PBC is a strong predictor of behavior compared to intention. For intention, attitude (coeff = 0.29, se = 0.13, *p* = 0.025), subjective norm (coeff = 0.37, se = 0.11, *p* = 0.001), and PBC (coeff = 1.29, se = 0.17, *p* = 0.000) are all significant predictors. PBC is the strongest predictor of intention, followed by subjective norm and attitude. The covariances and correlations among the constructs are also significant. All these parameters of the construct are positively correlated and interrelated. The model fit measures indicate a good fit: *χ*^2^ (78) = 2,597.468; CFI = 0.969; TLI = 0.958; RMSEA (95% CI) = 0.054 (0.043–0.064); SRMR = 0.042. These values suggest that the TPB model is well-suited to explain the relationships between the constructs and the observed behavior and intention.

**Table 3:** TPB model unstandardized coefficients (coeff), standard error (se), standardized coefficients (std), and *p*-values.

Endogenous variables	*R* ^2^	coeff	se	std	*p*
Behavior:	0.40				
Intention		0.16	0.06	0.24	**0.012**
Perceived behavioral control		0.54	0.14	0.42	**0.000**
Intention:	0.74				
Attitude		0.29	0.13	0.15	**0.025**
Subjective norm		0.37	0.11	0.19	**0.001**
Perceived behavioral control		1.29	0.17	0.66	**0.000**

Covariances and correlations	coeff	se	std	*p*
Attitude subjective norm	0.57	0.05	0.57	**0.000**
PBC subjective norm	0.48	0.05	0.48	**0.000**
PBC attitude	0.54	0.05	0.54	**0.000**

Model fit measures: *χ*^2^ (78) = 2,597.468; CFI = 0.969; TLI = 0.958; RMSEA (95% CI) = 0.054 (0.043–0.064); SRMR = 0.042.Bold values indicate statistical significance at a *p*<0.05.

Table 4 shows the findings of the extended TPB model, which integrates additional predictors such as sociodemographic variables, dietary habits, and physical activity levels. The model accounts for 78% of the variance in intention, 45% in behavior, 21% in attitudes, 12% in subjective norms, and 24% in PBC. The model demonstrates a good fit, as indicated by the following measures: *χ*^2^ (237) = 430.966; CFI = 0.955; TLI = 0.912; RMSEA (95% CI) = 0.045 (0.039–0.052); SRMR = 0.042.

**Table 4:** TPB-extended model unstandardized coefficients (coeff), standard error (se), standardized coefficients (std), and *p*-values.

	Intention				Behavior				Attitudes				SN				PBC			
*R* ^2^	0.78				0.45				0.21				0.12				0.24			
	coeff	se	std	*p*	coeff	se	std	*p*	coeff	se	std	*p*	coeff	se	std	*p*	coeff	se	std	*p*
Attitude	0.26	0.13	0.14	**0.042**																
SN	0.31	0.12	0.16	**0.010**																
PBC	1.32	0.21	0.71	**0.000**	0.71	0.26	0.61	**0.006**												
Intention					0.05	0.11	0.07	0.068												
Age									−0.01	0.01	−0.01	0.285	0.00	0.01	0.05	0.528	−0.00	0.01	−0.04	0.566
Gender									0.12	0.16	0.05	0.438	−0.10	0.16	−0.04	0.538	−0.00	0.16	−0.00	0.977
BMI									0.04	0.07	0.03	0.580	0.10	0.07	0.08	0.161	0.09	0.07	0.07	0.214
Marital status									0.17	0.15	0.07	0.264	−0.06	0.15	−0.03	0.674	−0.04	0.16	−0.02	0.809
Educational level									0.07	0.09	0.04	0.402	0.00	0.08	0.00	0.973	0.01	0.09	0.01	0.888
Place origin									0.05	0.06	0.04	0.433	0.01	0.06	0.01	0.872	−0.01	0.05	−0.01	0.818
Monthly income									0.01	0.07	0.01	0.844	−0.05	0.07	−0.04	0.465	0.02	0.07	0.02	0.742
Knowledge*									0.29	0.05	0.29	**0.000**	0.05	0.05	0.05	0.343	0.10	0.05	0.10	0.065
Family meals									0.19	0.05	0.23	**0.000**	0.07	0.05	0.09	0.150	0.04	0.05	0.05	0.425
F&V purchasing									0.05	0.06	0.05	0.387	0.01	0.06	0.01	0.823	0.16	0.07	0.14	**0.022**
F&V cooking/ preparing									0.07	0.06	0.08	0.217	0.11	0.06	0.14	0.053	0.10	0.06	0.11	0.089
Healthy diet									0.12	0.11	0.09	0.249	0.01	0.11	0.01	0.899	0.40	0.11	0.29	**0.000**
Balanced diet									−0.09	0.09	−0.07	0.284	0.07	0.09	0.06	0.424	−0.04	0.09	−0.03	0.616
Caloric diet									0.08	0.08	0.06	0.284	0.08	0.08	0.06	0.293	0.05	0.08	0.03	0.531
Intense PA									0.00	0.04	0.00	0.972	0.08	0.04	0.15	**0.031**	0.05	0.03	0.10	0.114
Moderate PA									0.04	0.03	0.09	0.154	0.08	0.03	0.17	**0.009**	0.05	0.03	0.11	0.073
Light PA									0.00	0.03	0.00	0.989	−0.01	0.03	−0.01	0.794	0.00	0.03	0.01	0.881
Any type of PA leading to increased heart beats									−0.07	0.07	−0.07	0.361	−0.14	0.07	−0.15	0.050	−0.01	0.07	−0.01	0.894

Model fit measures: *χ*^2^ (237) = 430.966; CFI = 0.955; TLI = 0.912; RMSEA (95% CI) = 0.045 (0.039–0.052); SRMR = 0.042.

SN, subjective norm; PBC, perceived behavioral control; PA, Physical Activity; F&V, Fruits and Vegetables.

*Knowledge regarding the WHO dietary consumption recommendations.Bold values indicate statistical significance at a *p*<0.05.

**Figure 1:**
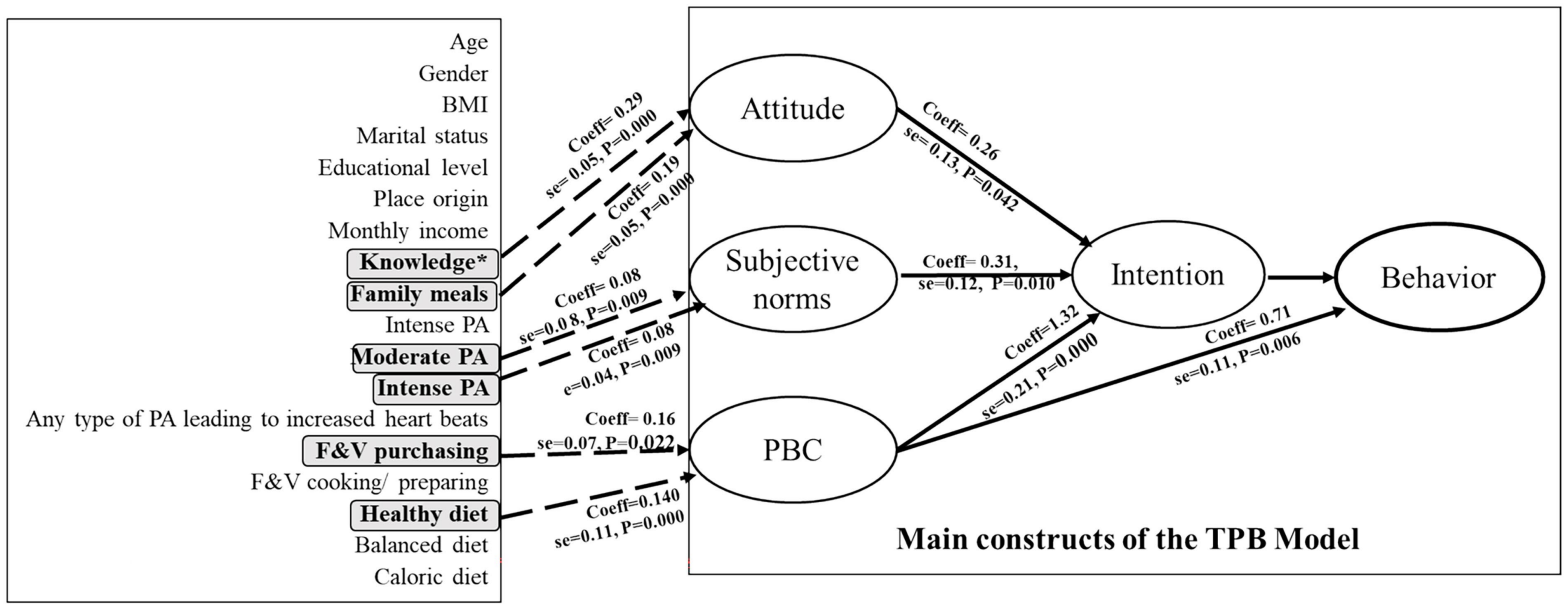
TPB-extended model and significant predictions. BMI, Body mass index; PA, Physical Activity; F&V, Fruits and Vegetables; *knowledge regarding the WHO dietary consumption recommendations.

The results indicated that intention is significantly predicted by attitude (coeff = 0.26, se = 0.13, *p* = 0.042), subjective norm (coeff = 0.31, se = 0.12, *p* = 0.010), and PBC (coeff = 1.32, se = 0.21, *p* = 0.000). Among these, PBC was the strongest predictor, followed by subjective norm and attitude. Notably, none of the sociodemographic variables significantly predict intention. For behavior, it is significantly predicted by PBC (coeff = 0.71, se = 0.26, *p* = 0.006). Attitudes toward fruits and vegetables consumption are significantly predicted by knowledge of WHO dietary recommendations (coeff = 0.29, se = 0.05, *p* = 0.000) and family meals (coeff = 0.19, se = 0.05, *p* = 0.000). Subjective norms are not significantly predicted by most sociodemographic or behavioral factors, except for intense physical activity (coeff = 0.08, se = 0.04, *p* = 0.031) and moderate physical activity (coeff = 0.08, se = 0.03, *p* = 0.009). In terms of PCB, this construct is significantly predicted by fruits and vegetables purchasing habits (coeff = 0.16, se = 0.07, *p* = 0.022) and perceptions of healthy dietary intake (coeff = 0.40, se = 0.11, *p* = 0.000). Main results are presented in Figure 1.

Table 5 highlights the interrelationships between sociodemographic variables, behavioral factors, and TPB constructs. Main findings indicate that among the TPB constructs, attitude is positively correlated with subjective norms (cov = 0.53, se = 0.05 corr = 0.48) and PBC (cov = 0.51, se = 0.06 corr = 0.48). Subjective norms is also positively correlated with PBC (cov = 0.41, se = 0.07, corr = 0.40), suggesting that social expectations and perceived control are interrelated. Knowledge regarding WHO dietary recommendations is positively correlated with family meals (covariance = 0.15, se = 0.05, correlation = 0.15) and fruits and vegetables purchasing (covariance = 0.18, se = 0.05, correlation = 0.18). In addition, family meals are positively correlated with fruits and vegetables purchasing (covariance = 0.23, se = 0.05, correlation = 0.24) and fruits and vegetables cooking/preparing (covariance = 0.25, se = 0.05, correlation = 0.27). Fruits and vegetables purchasing shows strong correlations with fruits and vegetables cooking/preparing (covariance = 0.29, se = 0.05, correlation = 0.30) and dietary intake (healthy wise) (covariance = 0.44, se = 0.04, correlation = 0.43). These findings highlight the role of family eating habits in promoting the consumption of fruits and vegetables.

**Table 5:** TPB-extended model, covariances, standard error, correlations.

Studied parameters	1	2	3	4	5	6	7	8	9	10	11	12	13	14	15	16	17	18	19	20	21
1. Attitude		0.53	0.51																		
		0.05	0.06																		
		0.48	0.48																		
	
2. SN			0.41																		
			0.07																		
			0.40																		
3. PBC																					
4. Age					ns	0.37	−0.60	0.23	−0.34	0.36	ns	0.12	0.25	0.22	0.24	0.24	ns	ns	ns	−0.14	ns
						0.04	0.04	0.05	0.05	0.05		0.05	0.04	0.05	0.04	0.04				0.05	
						0.39	−0.64	0.32	−0.36	0.36		0.12	0.24	0.27	0.23	0.21				−0.19	
5. Gender						0.20	ns	ns	ns	ns	ns	−0.18	ns	−0.37	−0.16	ns	0.11	ns	ns	ns	ns
						0.05						0.05		0.05	0.05		0.05				
						0.20						−0.19		−0.36	−0.16		0.11				
6. BMI							−0.27	0.16	ns	ns	ns	ns	ns	ns	−0.15	−0.10	0.18	ns	−0.12	−0.19	−0.12
							0.05	0.05							0.05	0.05	0.05		0.05	0.05	0.05
							−0.28	0.17							−0.13	−0.09	0.19		−0.12	−0.17	−0.12
7. Marital status								−0.25	0.25	−0.24	ns	−0.28	−0.15	−0.30	−0.11	−0.14	ns	0.12	0.12	0.19	0.15
								0.05	0.05	0.05		0.04	0.05	0.05	0.05	0.05		0.05	0.05	0.05	0.05
								−0.26	0.27	−0.23		−0.27	−0.15	−0.32	−0.12	−0.14		0.12	0.13	0.20	0.14
8. Educational level									ns	0.20	0.11	ns	0.09	ns	ns	ns	ns	ns	ns	ns	ns
										0.05	0.05		0.05								
										0.22	0.08		0.10								
9. Place origin										−0.18	ns	ns	ns	ns	−0.11	−0.10	ns	ns	ns	ns	ns
										0.05					0.05	0.05					
										−0.20					−0.13	−0.10					
10. Monthly income											0.11	0.12	0.14	ns	0.18	0.17	ns	ns	ns	ns	ns
											0.05	0.05	0.05		0.05	0.05					
											0.08	0.11	0.14		0.18	0.17					
11. Knowledge*												0.15	0.18	ns	ns	ns	0.10	0.10	ns	ns	ns
												0.05	0.05				0.05	0.05			
												0.15	0.18				0.09	0.11			
12. Family meals													0.23	0.25	0.18	0.19	0.10	ns	ns	ns	ns
													0.05	0.05	0.05	0.05	0.05				
													0.24	0.27	0.19	0.19	0.08				
13. F&V purchasing														0.29	0.44	0.42	−0.10	0.13	ns	0.11	0.11
														0.05	0.04	0.04	0.05	0.05		0.05	0.05
														0.30	0.43	0.43	−0.11	0.13		0.08	0.11
14. F&V Cooking/preparing															0.31	0.25	ns	ns	ns	ns	ns
															0.05	0.05					
															0.31	0.25					
15. Dietary intake (healthy wise)																0.72	−0.35	0.22	0.14	0.11	0.22
																0.03	0.05	0.05	0.05	0.05	0.05
																0.72	−0.34	0.24	0.12	0.08	0.21
16. Dietary intake (balanced wise)																	−0.25	0.18	0.14	ns	0.19
																	0.06	0.05	0.05		0.05
																	−0.26	0.20	0.13		0.20
17. Dietary intake (calorie wise)																		ns	−0.11	−0.13	−0.13
																			0.05	0.05	0.05
																			−0.11	−0.11	−0.13
18. Intense PA																			0.41	0.24	0.65
																			0.04	0.05	0.03
																			0.40	0.24	0.65
19. Moderate PA																				0.36	0.50
																				0.05	0.04
																				0.36	0.51
20. Light PA																					0.27
																					0.05
																					0.27
21. Any type of PA leading to increased heart beats																					

All covariances are significant at *p* < 0.005, ns, non-significant.

BMI, Body mass index; PA, Physical Activity; F&V, Fruits and Vegetables; *knowledge regarding the WHO dietary consumption recommendations.

Healthy diet is strongly correlated with balanced (covariance = 0.72, se = 0.03, correlation = 0.72), suggesting that individuals who perceive their diet as healthy also perceive it as balanced. Intense physical activity is positively correlated with moderate physical activity (covariance = 0.41, se = 0.04, correlation = 0.40) and light physical activity (covariance = 0.24, se = 0.05, correlation = 0.24). Moderate physical activity is strongly correlated with light physical activity (covariance = 0.36, se = 0.05, correlation = 0.36) and any type of physical activity leading to increased heartbeats (covariance = 0.50, se = 0.04, correlation = 0.51). These correlations suggest a consistent pattern of physical activity across different intensity levels, reflecting a holistic approach to maintaining an active lifestyle.

## 4 Discussion

The objective of this study is to conduct a comprehensive analysis of fruit and vegetable consumption among Saudi adults using the extended TPB. The findings provide critical insights into the determinants of dietary behavior in this population, emphasizing the roles of attitudes, SN, PBC, and additional behavioral and sociodemographic factors. The results confirm earlier findings indicating low levels of fruit and vegetable intake among Saudi adults (24, 26) and further highlight that both the average daily servings and the frequency of meeting the recommended five servings per day remain significantly inadequate. Overall, the results suggest that while participants hold positive attitudes, moderate perceived control, and intentions to consume fruits and vegetables, their actual consumption falls short of the recommended daily intake.

In line with the TPB framework, PBC emerged as the strongest predictor of both intention and behavior, followed by subjective norms and attitudes. This indicates that individuals’ beliefs about their capability, social expectations, and personal attitudes collectively shape their intentions to consume fruits and vegetables. These findings align with previous research, which highlights the central role of PBC in health-related behaviors, including dietary choices (11, 34). For example, studies in other populations have also identified PBC as the most significant predictor of fruit and vegetable consumption, while subjective norms and attitudes had a weaker direct impact on behavior (35, 36) (Kothe et al., 2012).

The comparison of *R*^2^ values between the basic TPB model (Table 3) and the TPB-extended model (Table 4) reveals important insights into the determinants of fruit and vegetable consumption among Saudi adults. The TPB-extended model explains slightly more variance in behavior (*R*^2^ = 0.45) compared to the basic TPB model (*R*^2^ = 0.40), indicating that the inclusion of additional predictors—such as sociodemographic factors, knowledge about WHO dietary recommendations, family meals, and physical activity—enhances the model’s ability to explain dietary behavior. However, the modest improvement suggests that while these factors contribute, the core TPB constructs (intention and PBC) remain the primary drivers of behavior (11, 34). Similarly, the extended model explains slightly more variance in intention (*R*^2^ = 0.78) compared to the basic model (*R*^2^ = 0.74), with knowledge and family meals strengthening the predictive power by influencing attitudes and subjective norms (18, 20). The extended model also provides insights into the variance explained for attitudes (*R*^2^ = 0.21), subjective norms (*R*^2^ = 0.12), and PBC (*R*^2^ = 0.24). These relatively low values suggest that these constructs are predicted by factors not fully captured in the model, such as cultural or contextual elements specific to Saudi Arabia (26, 28). For example, family influence and social norms may play a significant role in shaping dietary habits but are not explicitly accounted for in the current model. The enhanced explanatory power of the extended model is evident in the significant role of key predictors. Knowledge about WHO dietary recommendations strongly predicted attitudes (coeff = 0.29, se = 0.05, *p* < 0.001), highlighting the importance of education in shaping dietary intentions (37). Family meals were a significant predictor of attitudes (coeff = 0.19, se = 0.05, *p* < 0.001), emphasizing the role of family dynamics in promoting healthy eating (20). Additionally, moderate physical activity was associated with subjective norms (coeff = 0.08, se = 0.03, *p* = 0.009), suggesting a synergistic relationship between physical activity and dietary behavior (17).

In contrast, sociodemographic variables (e.g., age, gender, income) had minimal direct predictive effect behavior or intention, as reflected in their non-significant *p*-values. This suggests that dietary habits in Saudi Arabia may be more strongly determined by psychological and behavioral factors than by sociodemographic characteristics (16). The relatively low *R*^2^ values for subjective norms and attitudes further highlight the need to explore cultural and contextual factors, such as family influence and accessibility of fruits and vegetables, to enhance the model’s explanatory power (26).

From a practical perspective, the higher *R*^2^ for behavior in the extended model underscores the importance of addressing both psychological (e.g., PBC, intention) and practical factors (e.g., knowledge, family meals, physical activity) in interventions aimed at promoting fruit and vegetable consumption. The strong predictive effect of PBC (coeff = 0.71, se = 0.26, *p* = 0.006) on behavior highlights the need to enhance individuals’ confidence in their ability to consume fruits and vegetables, such as through cooking classes or meal planning workshops (38). Additionally, the significant role of family meals suggests that interventions should engage families and promote shared meals as a strategy to improve dietary habits (20).

The study found that physical activity levels, particularly intense and moderate activity, were positively associated with healthier dietary attitudes and behaviors. This aligns with research demonstrating a synergistic relationship between physical activity and healthy eating. For example, a study in Canada found that individuals who engaged in regular physical activity were more likely to consume fruits and vegetables (17). These findings suggest that interventions promoting both physical activity and healthy eating may be more effective than those targeting diet alone, as they address multiple health behaviors simultaneously.

This study has several strengths, including the use of an extended TPB model to explore a wide range of predictors and the inclusion of respondents from the different age groups and regions of Saudi Arabia. However, there are also limitations. The cross-sectional design limits the ability to establish causal relationships, and self-reported data may be subject to bias. Future research should consider longitudinal designs and objective measures of the reported parameters.

## 5 Conclusion

This study provides valuable insights into the factors influencing vegetable and fruit consumption among Saudi adults. Utilizing an extended TPB model, the findings highlight the importance of PBC, knowledge, family habits, and physical activity in shaping dietary behavior. Interventions that address these factors while considering sociodemographic contexts are likely to be most effective in promoting healthy eating habits in this population. By fostering a supportive environment and empowering individuals with the skills and confidence to make healthier choices, public health initiatives can contribute to reducing the burden of diet-related diseases in Saudi Arabia. Despite its strengths—including a diverse sample and comprehensive theoretical framework—this study has some limitations. The cross-sectional design restricts the ability to infer causality, and reliance on self-reported data may introduce response bias. Future research should employ longitudinal designs and incorporate objective measures of dietary intake and related behaviors to validate and expand upon these findings. Addressing these limitations will help refine strategies aimed at reducing the burden of diet-related diseases in Saudi Arabia and beyond.

## Data Availability

The raw data supporting the conclusions of this article will be made available by the authors, under reasonable request.
